# Does implementation of an enhanced recovery after surgery program for hip replacement improve quality of recovery in an Australian private hospital: a quality improvement study

**DOI:** 10.1186/s12871-018-0525-5

**Published:** 2018-06-13

**Authors:** Nicole Lay Tin Tan, Justin Lindley Hunt, Stella May Gwini

**Affiliations:** 1 Honorary Clinical Fellow, Faculty of Medicine, Dentistry and Health Sciences, The University of Melbourne, Melbourne, Australia; 2 Epworth HealthCare, 89 Bridge Rd, Richmond, Vic 3121 Australia

**Keywords:** Enhanced recovery after surgery, Fast-track program, ERAS, Quality of recovery, Quality improvement, Observational study, Hip arthroplasty, Hip replacement

## Abstract

**Background:**

Enhanced recovery after surgery programs may improve recovery and reduce duration of hospital stay after joint replacement surgery. However, uptake is incomplete, and the relative importance of program components is unknown. This before-and-after quality improvement study was designed to determine whether adding ‘non-surgical’ components, to pre-existing ‘surgical’ components, in an Australian private healthcare setting, would improve patient recovery after total hip replacement.

**Methods:**

We prospectively collected data regarding care processes and health outcomes of 115 consecutive patients undergoing hip replacement with a single surgeon in a private hospital in Melbourne, Australia. Based on this data, a multidisciplinary team (surgeon, anesthetists, nurse unit managers, physiotherapists, perioperative physician) chose and implemented 12 ‘non-surgical’ program components. Identical data were collected from a further 115 consecutive patients. The primary outcome measure was Quality of Recovery-15 score at 6 weeks postoperatively; the linear regression model was adjusted for baseline group differences.

**Results:**

The majority of health outcomes, including the primary outcome measure, were similar in pre- and post-implementation groups (quality of recovery score, pain rating and disability score, at time-points up to six weeks postoperatively). The proportion of patients with zero oral morphine equivalent consumption at six weeks increased from 57 to 80% (RR 1.34, 95% CI 1.13, 1.58). Mean (SD) length of hospital stay decreased from 5.94 (5.21) to 5.02 (2.46) days but was not statistically significant once adjusted for baseline group differences.

Four of ten measurable program components were successfully implemented. Antiemetic prophylaxis increased by 53% (risk ratio [RR] 95% confidence interval [CI] 1.16, 2.02). Tranexamic acid use increased by 41% (RR 95% CI 1.18, 1.68). Postoperative physiotherapy treatment on the day of surgery increased by 87% (RR 95% CI 1.36, 2.59). Postoperative patient mobilisation ≥ three metres on the day of surgery increased by 151% (RR 95% CI 1.27, 4.97).

**Conclusions:**

Implementation of a full enhanced recovery after surgery program, and optimal choice of program components, remains a challenge. Improved implementation of non-surgical components of a program may further reduce duration of acute hospital stay, while maintaining quality of recovery.

**Trial registration:**

Australian New Zealand Clinical Trials Registry (ACTRN12615001170516), 2.11.2015 (retrospective).

## Background

‘Enhanced recovery after surgery’ (ERAS) programs have shown improvements in patient recovery after joint replacement, as measured by hospital length of stay, improved early mobilisation and patient satisfaction, without adversely affecting surgical outcomes [1–5]. Teams from Australian and New Zealand hospitals have recently published similar results [6–8].

However, wider uptake of comprehensive ERAS programs has been slow or incomplete. This is an example of challenges in ‘bridging the second translation gap’ [9], in which efficacious treatments in a research setting must be demonstrated to be effective in daily practice. In fact, Kehlet, the originator of the ERAS concept in the early 1990s, recently stated that ‘in most of the surgical world, enhanced recovery principles remain either foreign or unimplemented’ [10].

One reason may be the requirement for multidisciplinary collaboration, and organisational factors that delay change [11]. These are likely to be particular challenges in large private healthcare organizations, however published data is sparse. In these settings, multiple, organisationally independent surgeons, anesthetists and physicians intersect in varying combinations with hospital nurses and allied health staff on several subspecialty wards. For example, our surgical theatre team (surgeon JH) routinely utilizes surgical components of an ERAS program: a minimally invasive anterior approach for total hip replacement (THR), local infiltration analgesia, cell saver, and no wound drains or urinary catheters. These components are simple to institute in the contained theatre environment. However, anecdotal evidence suggested that variations in care and management processes between different anesthetists, physiotherapists and orthopedic wards affected patient outcomes such as postoperative nausea and vomiting (PONV), pain and mobilization.

A second reason may be that there is conflicting or confusing evidence for some common components of ERAS programs for joint replacement. For example, preoperative education has been shown to reduce length of stay in small studies with voluntary participants [12, 13] however a Cochrane review did not show an improvement in patient anxiety or surgical outcomes [14]. Preoperative carbohydrate loading is recommended in guidelines for enhanced recovery for colorectal surgery [15], but there is limited evidence for its use in joint arthroplasty [16, 17]. Large meta-analyses have shown neuraxial and regional anesthesia to provide equivalent [18, 19] or better [20] clinical outcomes compared with general anesthesia. Benefits of restricted or goal-directed fluid therapy may be less in joint arthroplasty than colorectal surgery [21]; arthroplasty-specific studies are ongoing. In comparison, the benefits of multimodal analgesia [22, 23], PONV prophylaxis [24], normothermia [25, 26], tranexamic acid [27] and early mobilization [28, 29] are well-described in arthroplasty surgery.

In 2016 46,000 hip replacements were performed in Australia [30] - 59% in the private sector [31, 32] - therefore even small improvements in postoperative recovery have the potential for wide-ranging benefits. The objective of this study was to determine whether addition of non-surgical components of an ERAS program would improve patient recovery after THR. The hypothesis was that patients undergoing THR with surgical and non-surgical components of an ERAS program would have an improved recovery, measured by Quality of Recovery-15 (QoR-15) score at 6 weeks, compared with those undergoing THR with only surgical components of an ERAS program.

## Methods

The STROBE guidelines for reporting observational studies [33] have been followed.

### Design and setting

This prospective, before-and-after interventional study took place in a 500-bed, university-affiliated, non-profit private hospital in metropolitan Melbourne, Australia, from January 2015 to August 2016. JH performs approximately 220 THR a year. Six regular anesthetists, ten locum anesthetists, three surgical assistants, one regular physician and two locum physicians were involved in the study period. Patients were admitted to one of four inpatient wards. Inpatient physiotherapy was provided by a team of six regular and eight locum physiotherapists.

This study assessed pre-existing care processes and outcomes of patients undergoing THR with surgical components of an ERAS program. Based on pre-implementation data, a multidisciplinary team chose additional non-surgical components, then implemented a full ERAS program. We then assessed the impact of that program.

### Pre-implementation phase

In the pre-implementation phase, data on patient demographics, perioperative care processes and postoperative outcomes up to 6 weeks were collected from 115 consecutive patients undergoing THR from 27 January 2015 to 18 September 2015. Patients were provided with written information regarding surgery and recovery. THR was performed with an anterior surgical approach. Local infiltration analgesia was performed with 100-200 ml of 0.2% ropivacaine. Cell salvage was used for all cases, wound drains were not utilized and urinary catheters were not inserted unless for urinary incontinence/retention. Anesthetic and analgesic techniques were at the anesthetist’s discretion. Cefazolin 2 g was administered intravenously 5–15 min before skin incision; cefazolin 1 g intravenously was administered at 8-hourly intervals for 48 h. Enoxaparin 40 mg was administered subcutaneously 6–8 h after neuraxial anesthesia, or during surgery if neuraxial anesthesia was not used, and continued daily for 21 days.

Pre-implementation physiotherapy was not standardised but typically included mobilization on day 0 or day 1 with progression towards independent transfers, ambulation with crutches and stairs, as well as a graded exercise program to improve lower limb range of movement and strength. Inpatient education was provided regarding care transfers, a home exercise program and functional progression after discharge.

### ERAS program design

The program items implemented are listed in Table 1. Successful implementation of quality improvement programs relies heavily on uptake by end-users, thus their engagement in program design is essential. For example, no epidurals or regional blocks were used in the pre-implementation phase, therefore we were concerned that their implementation in the ERAS program would not be successful. Instead, emphasis was placed on the use of anesthetic techniques to enable early oral intake and mobilization, such as spinal anesthesia and sedation. Tranexamic acid 1 g and at least one anti-emetic was administered intraoperatively.

**Table 1:** Program items implemented

1.^a^Written multidisciplinary preoperative patient information.	
2. Reduction in preoperative duration of fasting (time from cessation of clear fluids to spinal or induction of general anesthesia, whichever came first). Reduction in postoperative duration of fasting (time from completion of suturing to first intake of clear fluids)	
3. Increase in spinal anesthesia	
4. Decrease in general anesthesia (use of laryngeal mask airway, endotracheal tube or bispectral index < 60)	
5. Intraoperative antiemetic prophylaxis (at least 1 antiemetic)	
6. Tranexamic acid (1 g intravenously at commencement of surgery)	
7. Intraoperative forced air warmer and fluid warming	
8. Oral multimodal analgesia	
9. Cessation of intravenous fluid on day 1	
10. Postoperative physiotherapy assessment on day of surgery	
11. Postoperative independent mobilisation ≥3 m on day of surgery	
12.^a^Predefined discharge criteria	

^a^degree of implementation of items 1 and 12 was not measured

Unless contraindicated, multimodal analgesia consisted of regular oral paracetamol, a non-steroidal anti-inflammatory or cox_2_ inhibitor, slow release oxycodone, and immediate release oxycodone as required. Intravenous opioid analgesia was discouraged. Tramadol and gabapentinoids were prescribed at the anesthetist’s discretion. Pre-operative analgesia was to be continued post-operatively. Intravenous fluid was to cease on postoperative day 1.

The orthopedic physiotherapy team chose the outcome measure (the 10 meter walk test [10MWT]), and designed the mobilization plan. After physiotherapist assessment on the day of surgery, patients aimed to mobilize on a walking frame around their room and to the bathroom. On day 1, they aimed to mobilize on elbow crutches, sit out of bed and shower. On day 2, they aimed to independently mobilize more than 50 m with elbow crutches and independently perform self-care activities. On day 3, they aimed to independently transfer and mobilize more than 100 m, and have a clear understanding of post-discharge progression of the ambulation and exercise program. If applicable, they were to be independent with stairs and car transfers.

The orthopaedic nurse unit managers, in conjunction with the team, defined the discharge criteria. Patients were fit for discharge when medically stable, pain was controlled on oral analgesia, the wound was clean and dry, they had recommenced (or had a plan to recommence) usual medications, were able to administer thromboembolic prophylaxis, had returned to (or had a management plan for) usual voiding and bowel patterns, met physiotherapy discharge criteria, and post-discharge supports were in place if required. To be considered ready for discharge, all patients had to meet all discharge criteria.

Only existing or accessible resources were used for this study. For example, our hospital does not have a multidisciplinary pre-admission clinic, so attendance was not included as a program component. In addition to usual written information regarding surgery, patients were provided with written information regarding less restrictive preoperative fasting instructions (clear fluids allowed up to 2 h preoperatively), anesthesia techniques, oral multimodal analgesia, inpatient physiotherapy plan, and information for discharge planning on day 3.

### Post-implementation phase

Following a 1-month implementation period, post-implementation data were collected from a further 115 consecutive patients undergoing THR from 16 October 2015 to 22 June 2016, to measure degree of implementation and outcomes of the program. The team decided a priori that it was not feasible to measure ‘successful’ patient education (item 1) and time of readiness for discharge (item 12). No additional funding for clinical resources was provided.

### Outcome measures

There is no consensus on a core set of measures for an ERAS program [34], therefore we chose patient-centred outcome measures. The primary outcome measure was QoR-15 at 6 weeks postoperatively. QoR-15 is a commonly used, well-validated, multidimensional patient-reported quality of recovery scale, with 15 items which assess the domains of pain, physical comfort, physical independence, emotions and psychological support [35]. Scores from the 15 items are summed to form a composite score that ranges from 0 (extremely poor recovery) to 150 (excellent recovery).

Other outcomes were the degree of package implementation; QoR-15 on postoperative day 1 and 2; highest pain score (Numeric Rating Scale [NRS; 0–10, 0: no pain, 10: worst imaginable] at rest and on movement, in the previous 24 h) on day 1, day 2 and week 6; oral morphine equivalent (OME) consumption (mg/day) [36] on day 1, day 2 and week 6; 10 m walk test (10MWT; time taken to walk 10 m as measured by physiotherapist) on day 1, 2 or 3; length of acute hospital stay (from day of procedure to day of discharge); World Health Organization Disability Assessment Schedule 2.0 (WHODAS 2.0) [37] at 6 weeks; unplanned hospital readmission and major complication rate (wound or prosthesis infection, joint dislocation, deep venous thrombosis, pulmonary embolus, myocardial ischemia requiring hospital admission or intervention, transient ischaemic attack, stroke, new kidney disease) up to week 6.

### Data sources

During admission, data was collected from the patient and hospital medical records, by a hospital research nurse. Intraoperative data was collected by the treating anesthetist. Data from discharge to week 6 was supplied by the patient when telephoned by the research nurse at week 6; reasons for readmission and return to theatre were confirmed through hospital records.

### Statistical analysis

Sample size estimation conducted before study commencement indicated that 100 patients per group were required to detect a 6-point difference in QoR-15 at 6 weeks, with 0.05 level of statistical significance and 80% power. We recruited an additional 15 patients per group to allow for loss to follow up at 6 weeks.

Summary statistics of dimensional variables are presented as means and standard deviations for normally distributed data, and medians and interquartile ranges for skewed data. Univariate comparisons between groups were conducted using simple linear regression for normally distributed data and Wilcoxon rank-sum test for skewed data. Categorical variables are reported as frequencies and percentages, and between group comparisons were conducted using Chi-square tests. Comparison was not performed for variables with very low frequencies.

QoR-15, NRS and WHODAS 2.0 were analysed using linear regression models while 10MWT and OME were analysed using median regression. Length of stay was analysed using negative binomial regression, an alternative method to Poisson regression for analysing count data, when count data is over-dispersed i.e. when variance is larger than the mean. All analyses were conducted using Stata Statistical Software [38]. Regression models were adjusted for respiratory disease, preoperative anemia and history of PONV, because there was a potential clinically significant difference between pre- and post-implementation groups.

To reduce the risk of selection bias and increase generalizability of our findings, we recruited consecutive participants, rather than a selected sample. Planned subgroup analysis was undertaken for patients with osteoarthritis who underwent primary, unilateral, anterior THR; this group was expected to have an improved recovery compared with those undergoing THR for other pathology, or bilateral or revision THR.

## Results

The pre-implementation group consisted of 115 consecutive patients. Of 121 potential participants in the post-implementation phase, 5 patients were not approached because they were enrolled in the pre-implementation phase, and 1 patient was not enrolled because they were cognitively unable to complete the primary outcome measure.

There was 100% follow-up to hospital discharge in both the pre- and post-implementation phases. At the final 6-week follow-up, 3 patients in the pre-implementation group could not be contacted, and 2 declined further participation. In the post-implementation group, 1 patient provided QoR-15 score but not pain score.

Pre- and post-implementation groups were similar with regard to age, sex, BMI (body mass index), ASA (American Society of Anesthesiologists Physical Status classification), smoking status, known diabetes mellitus, coronary artery disease and chronic kidney injury (Table 2). Pre- and post-implementation groups were similar with regard to surgical characteristics (Table 3).

**Table 2:** Preoperative patient characteristics of pre-implementation and post-implementation groups

Characteristics	Pre-implementation *n* = 115	Post-implementation *n* = 115	*p*-value
Age			
Mean (SD)	63.9 (10.27)	64.6 (10.44)	0.615
Sex: n(%)			
Female	75 (65.2)	78 (67.8)	0.675
Male	40 (34.8)	37 (32.2)	
BMI			
Mean (SD)	28.89 (5.96)	27.49 (5.57)	0.068
ASA: n (%)			
I	25 (22.5)	29 (25.2)	0.732
II	52 (46.9)	56 (48.7)	
III	34 (30.6)	30 (26.1)	
Smoking status: n (%)			
Non-smoker	106 (92.2)	106 (92.2)	> 0.999
Medical comorbidities: n(%)			
Known diabetes mellitus	5 (4.4)	3 (2.6)	0.722
Coronary artery disease	9 (7.8)	9 (7.8)	> 0.999
Respiratory disease	24 (20.9)	10 (8.7)	0.015
Anaemia^a^	12 (10.4)	4 (3.5)	0.067
History of PONV	35 (30.4)	19 (16.7)	0.014
Chronic kidney injury^b^	4 (3.5)	1 (0.9)	0.175
QoR-15 score			
Mean (SD)	113.94 (17.85)	115.70 (17.17)	0.447
Worst NRS score mean: (SD)			
Rest	3.81 (2.73)	3.58 (2.79)	0.527
Movement	5.90 (2.47)	5.92 (2.61)	0.967
OME consumption (mg/day)			
Median (Q1, Q3)	0 (0, 0)	0 (0, 0)	0.626

Abbreviations: *ASA* American Society of Anesthesiologists Physical Status classification, *NRS* numerical rating scale, *OME* oral morphine equivalents, *PONV* postoperative nausea and vomiting, *QoR-15* Quality of Recovery-15 score, *SD* standard deviation

^a^Anaemia: < 128 g/l for men, < 115 g/l for women

^b^Chronic kidney injury: creatinine > 0.13umol/l

**Table 3:** Surgical characteristics of patients

Characteristics	Pre-implementation	Post-implementation	*p*-value
Surgical pathology: n (%)			
Osteoarthritis	106 (92.2)	109 (94.8)	0.423
Type of surgery: n (%)			
Primary	113 (98.3)	112 (97.4)	> 0.999
Unilateral	112 (97.4)	113 (98.3)	> 0.999
Anterior approach	114 (99.1)	114 (99.1)	> 0.999
Duration of surgery (mins)			
Mean (SD)	67.2 (22.77)	65.3 (28.18)	0.274

### Outcomes

**Fig. 1:**
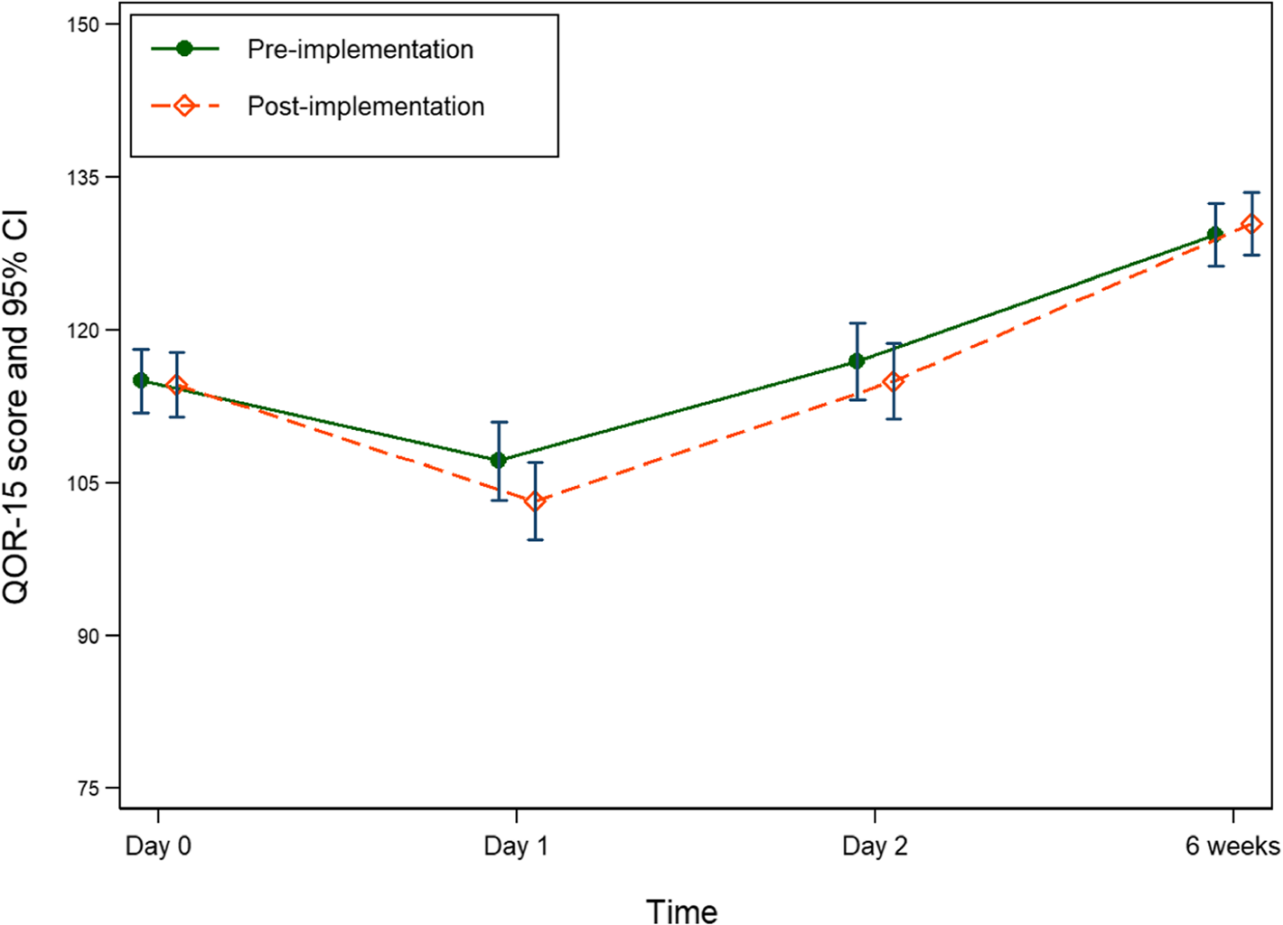
QoR-15 score (primary outcome measure) for pre- and post-implementation groups at 4 time-points

All outcome comparisons were adjusted for baseline group differences, that is pre-existing respiratory disease, anaemia and history of PONV (Table 4). There was no significant difference in mean QoR-15 score between pre- and post-implementation groups at the measured time-points (Fig. 1). Subgroup analysis of those with osteoarthritis who underwent primary, unilateral, anterior THR (103 in pre- and 105 in post-implementation groups) showed no significant differences between groups. Mean QoR-15 score on day 2 was similar to the preoperative score (day 0) for both pre- and post-implementation groups.

**Table 4:** Program outcome results

Outcome	Pre-implementation	Post-implementation	Difference or RoM (95% CI)	*p*-value
QoR-15: mean (SD)^a^				
Day 1	106.13 (22.86)	103.83 (21.31)	Diff −3.96 (−9.41, 1.49)	0.154
Day 2	116.13 (21.73	115.04 (20.96)	Diff −1.94 (−7.25, 3.38)	0.476
Week 6 (primary outcome)	128.87 (17.10)	131.14 (13.50)	Diff 1.09 (−3.31, 5.49)	0.628
Worst NRS score: mean (SD)^b^				
Day 1 (rest)	3.73 (2.92)	4.14 (3.14)	Diff 0.33 (−0.45, 1.11)	0.401
Day 1 (movement)	5.35 (2.60)	5.46 (2.81)	Diff 0.14 (−0.53, 0.81)	0.681
Day 2 (rest)	2.54 (2.27)	3.00 (2.67)	Diff 0.43 (−0.20, 1.07)	0.183
Day 2 (movement)	4.52 (2.61)	4.78 (2.58)	Diff 0.28 (−0.39, 0.96)	0.414
Week 6 (rest)	1.18 (1.88)	0.92 (1.38)	Diff −0.19 (− 0.66, 0.29)	0.444
Week 6 (movement)	1.76 (2.25)	1.89 (2.21)	Diff 0.19 (−0.46, 0.85)	0.562
OME consumption (mg/day): mean (SD)^c^				
Day 1	75 (45, 120)	75 (46, 105)	Diff −5.0 (−20.21, 10.21)	0.518
Day 2	45 (30, 75)	50 (30, 75)	Diff 0 (−11.94, 11.94)	> 0.999
Week 6	0 (0, 15)	0 (0, 0)	Diff 0 (− 10.90, 10.90)	> 0.999
10MWT (minutes): median (Q1; Q3)				
Day 3	0.40 (0.28; 0.71)	0.47 (0.26; 0.60)	Diff 0.04 (−0.06, 0.15)	0.440
Duration of hospital stay (days): mean (SD)				
Entire group	5.94 (5.21)	5.02 (2.46)	RoM 0.89 (0.74, 1.07)	0.212
Unadjusted comparison			RoM 0.84 (0.70, 1.02)	0.072
Subgroup	5.91 (5.35)	4.89 (1.97)	RoM 0.86 (0.71, 1.04)	0.114
Unadjusted comparison			RoM 0.83 (0.68, 1.00)	0.051
WHODAS 2.0 score: mean (SD)				
Week 6	18.17 (7.06)	17.97 (6.37)	Diff 0.40 (−1.42, 2.22)	0.663
Unplanned hospital readmission: n(%)	5 (4.35)	8 (6.96)		

Abbreviations: *NRS* numeric rating scale, *OME* oral morphine equivalents, *Q1* 25th percentile, *Q3* 75th percentile, *QoR-15* Quality of Recovery-15, *RoM* ratio of means

^a^adjusted for pre-operative QoR-15, respiratory disease, preoperative anaemia and history of PONV.

^b^adjusted for pre-operative NRS score, respiratory disease, preoperative anaemia and history of PONV

^c^adjusted for pre-operative OME, respiratory disease, preoperative anaemia and history of PONV

Between pre- and post-implementation groups, there was no significant difference in average NRS pain scores, OME consumption or WHODAS 2.0 score at any time-point. The proportion of patients with zero OME consumption at week 6 increased from 56.6 to 80.0% (RR 1.34, 95% CI 1.13, 1.58). The most common day on which the 10MWT was performed was postoperative day 3 (61 and 65 patients in pre- and post-implementation groups respectively). There was no significant difference between pre- and post-implementation groups.

The mean length of hospital stay decreased in the post-implementation group compared with the pre-implementation group, but this result was not statistically significant when adjusted for baseline group differences. The variance in length of stay was significantly smaller in the post-implementation group (*p* = 0.002).

Within the first six weeks postoperatively, five patients in the pre-implementation group had an unplanned readmission to hospital (two with hip fractures requiring revision hip surgery, one with surgical infection requiring wound washout, one for treatment of pneumonia and one for pain management). Eight patients in the post-implementation group had an unplanned readmission (two with hip fractures requiring revision hip surgery, one for acetabular revision, one with urinary retention requiring prostate surgery, one with allergy to surgical skin preparation solution, two treated with intravenous antibiotics for possible wound infection and one for pain management). There were no other reported major medical complications.

### Program implementation

Compared with pre-implementation (Table 5), there was a significant increase in the post-implementation proportion of patients administered intraoperative antiemetic prophylaxis (item 4), and a significant decrease in the proportion of patients with PONV on day 1. Administration of tranexamic acid significantly increased (item 5) but was not associated with a reduction in blood loss or allogeneic blood transfusion. Significantly more patients received postoperative physiotherapy assessment (item 9) and mobilised ≥3 m independently (item 10) on the day of, and the day after, surgery.

**Table 5:** Degree of program implementation

Program item		Pre-implementation	Post-implementation	Difference or RR (95% CI)	*p*-value
1. Duration of fasting (hours): median (Q1; Q3)					
Preoperative		11.8 (9.92; 13.42)	10.9 (7.67; 13.00)	Diff −0.83 (− 1.96, 0.29)	0.147
Postoperative		1.25 (0.82; 3.00)	1.48 (0.80; 3.02)	Diff 0.25 (− 0.30, 0.80)	0.368
2. Spinal anesthesia: n (%)		101 (88.6)	103 (89.6)	RR 1.01 (0.92, 1.11)	0.815
3. General anesthesia: n (%)		55 (47.7)	44 (38.9)	RR 0.81 (0.60, 1.10)	0.180
4. Intraoperative antiemetic prophylaxis: n (%)		45 (39.1)	64 (55.7)	RR 1.53 (1.16, 2.02)	0.003
PONV day 1		44 (38.3)	30 (26.6)	RR 0.57 (0.43, 0.76)^a^	< 0.001
PONV day 2		21 (18.3)	22 (19.1)	RR 0.84 (0.51, 1.40)^a^	0.501
5. Intraoperative tranexamic acid: n (%)		68 (59.1)	96 (83.5)	RR 1.41 (1.18, 1.68)	< 0.001
Intraoperative blood loss (ml): median (Q1; Q3)		250 (150; 450)	250 (150; 475)	Diff 0.00 (−66.2 to 66.2)	> 0.999
Blood transfusion day 1		3 (2.6)	0 (0.0)		
Blood transfusion day 2		4 (3.5)	2 (1.7)		
6. Forced air warming: n (%)		113 (98.3)	114 (99.1)	RR 1.01 (0.98, 1.04)	0.562
Fluid warming: n (%)		110 (95.7)	104 (90.4)	RR 0.95 (0.88, 1.02)	0.123
PACU temperature (°C): mean (SD)		35.6 (0.59)	35.6 (0.52)	Diff −0.04 (−0.18, 0.11)	0.611
7. Oral analgesia: n (%)					
Paracetamol	Day 1	109 (94.8)	114 (99.1)	RR 1.05 (1.00, 1.10)	0.057
	Day 2	107 (93.0)	114 (99.1)	RR 1.06 (1.01, 1.12)	0.019
NSAIDs/cox_2i_	Day 1	50 (43.5)	72 (62.6)	RR 1.44 (1.12, 1.85)	0.005
	Day 2	59 (51.3)	65 (56.5)	RR 1.10 (0.87, 1.40)	0.429
Tramadol	Day 1	37 (32.2)	41 (35.7)	RR 1.11 (0.77, 1.59)	0.579
	Day 2	27 (23.7)	23 (20.0)	RR 0.84 (0.52, 1.38)	0.502
Gabapentinoids	Day 1	22 (19.1)	34 (29.6)	RR 1.55 (0.97, 2.47)	0.070
	Day 2	19 (16.7)	31 (27.0)	RR 1.62 (0.97, 2.69)	0.065
Intravenous analgesia: n(%)					
Opioid PCA	Day 1	5 (4.4)	5 (4.4)		
	Day 2	2 (1.7)	1 (0.87)		
Ketamine	Day 1	1 (0.09)	2 (1.7)		
	Day 2	1 (0.09)	1 (0.09)		
Epidural or nerve block: n(%)		0 (0.0)	4 (3.5)		
8. Cessation of intravenous fluid on day 1: n(%)		96 (83.5)	93 (80.9)	1.13 (0.65, 1.97)	0.673
9. Postoperative physiotherapy assessment: n (%)					
Day 0		34 (29.6)	66 (57.4)	RR 1.87 (1.36, 2.59)	< 0.001
Day 1		76 (66.1)	113 (98.3)	RR 1.44 (1.26, 1.63)	< 0.001
Day 2		113 (98.3)	114 (99.1)	RR 1.00 (0.98, 1.02)	0.995
10. Postoperative mobilisation ≥ 3 m: n (%)					
Day 0		10 (9.0)	26 (22.6)	RR 2.51 (1.27, 4.97)	0.008
Day 1		76 (68.5)	94 (81.7)	RR 1.19 (1.02, 1.39)	0.024
Day 2		99 (89.2)	112 (97.4)	RR 1.09 (1.02, 1.17)	0.016

Abbreviations: *PACU* Post-anesthesia care unit, *PCA* Patient controlled analgesia, *Q1* 25th percentile, *Q3* 75th percentile

^a^adjusted for history of PONV

Use of oral multimodal analgesia (paracetamol, NSAIDs/cox_2i_) did not increase in a clinically significant manner, except for NSAIDS/cox_2i_ on day 1. There was no change in duration of pre- or post-operative fasting, use of spinal or general anesthesia, rates of intraoperative patient warming or cessation of intravenous fluid by day 1.

## Discussion

There was no significant difference in the primary outcome (quality of recovery score) or other measured outcomes, between the pre- and post-implementation groups.

Four of ten package items were successfully implemented. Two items were simple to administer (intraoperative antiemetic prophylaxis and tranexamic acid). The remaining two were achieved by improved coordination of team workload and cessation of weekend surgery (increased physiotherapy treatment and increased early mobilisation).

Some package items were not fully implemented. Despite clear written instructions, duration of preoperative fasting did not decrease as expected; inconsistent verbal advice from hospital staff and previous patient experience of fasting for surgery are likely to be responsible.

There was no significant change in the proportion of patients receiving general anesthesia or oral multimodal analgesia. This may be due to medical contraindications, such as use of NSAIDs in the setting of kidney disease, or patient preference for a particular technique, such as general anesthesia. Because of our institution’s ‘independent doctor’ model, we used ‘recommended’ rather than ‘compulsory’ items in the ERAS program. Thus, anesthetist preference for a different anesthetic and analgesic technique may also be a contributor.

Though not specifically included in the ERAS program, use of gabapentinoids increased in the post-implementation group (RR 1.55 [0.97, 2.47]). In Australia’s Pharmaceutical Benefits Scheme, gabapentinoids are listed for use only in neuropathic pain (gabapentin and pregabalin) or epilepsy (gabapentin). This means that their use in a postoperative setting is considered by the relevant government authority to be unsupported by strong evidence and incurs additional financial costs to the patient. For this reason, we did not specifically include gabapentinoids in our ERAS program.

There was no change in the implementation of three items (spinal anesthesia, intraoperative patient warming, and cessation of intravenous fluid on day 1) which had high pre-implementation rates of 89, 96 and 84% respectively. Though we wished to improve up-take of these items, with the benefit of hindsight this was unlikely to occur. Nonetheless, these evidence-based components will remain in future ERAS audits.

The choice of non-surgical package items may not be optimal, as there is limited or conflicting evidence for some ERAS components such as preoperative patient education and optimal anaesthetic technique. The effect of unmeasured package items (patient education, defined discharge criteria) cannot be examined. We attempted to ameliorate the impact of locums and staff changes by displaying and promulgating written guidelines; it is likely that unfamiliarity with the ERAS program resulted in a degree of non-adherence.

Finally, the study’s outcomes may be influenced by factors beyond our control, such as availability of rehabilitation beds. Unfortunately, we did not have the resources to precisely time each patient’s readiness for discharge, and reasons for non-discharge. Though the shortage of rehabilitation beds is reportedly not as severe in our hospital compared with others, delayed discharge from acute care still occurs for this reason. Conversely, if a rehabilitation bed is pre-booked for a certain day, patients cannot be discharged earlier. Notably, the return of mean QoR-15 score to pre-operative levels by day 2 suggests that patients may be ready for discharge before day 5.

This study’s strengths are the enrolment of consecutive patients in order to reduce the likelihood of selection bias and increase generalizability of our findings; we did not exclude patients with comparatively slower expected recovery due to demographic, medical or surgical factors. A multidisciplinary approach was used to design the program, using existing clinical and organisational resources. We also measured the degree to which each program item was implemented, and the chosen recovery outcome measures were patient-centred.

Though well-recognised in this field, this study’s prospective before-and-after design is its main limitation. A prospective parallel-group design, randomised or otherwise, was not feasible in this setting. We therefore cannot exclude the impact of change in season, or the ‘self-improving’ tendency of systems, nor regression to the mean.

Comparison of ERAS programs between countries and institutions is hindered by the variability of program components, which are tailored to locally available resources. Published studies of ERAS programs that demonstrate a reduced length of stay have been undertaken overseas, combine knee and hip replacement groups, or incorporate both surgical and non-surgical components of a program. Our study is the first to specifically report patient-centred outcomes, and address the challenges of implementing an ERAS program for THR in an Australian private healthcare setting.

## Conclusions

Implementation of a full enhanced recovery after surgery program, and optimal choice of program components, remains a challenge. Improved implementation of non-surgical components of a program may further reduce duration of acute hospital stay, while maintaining quality of recovery. Assessing the implementation and outcomes of clinician-driven ERAS programs on a larger scale, for example those of multiple surgical teams within or across large institutions, will assist in determining the relative importance of individual components of an ERAS program, as well as more effective implementation strategies.

## Abbreviations

10MWT: 10 meter walk test
ASA: American Society of Anesthesiologists physical status classification
BMI: body mass index
CI: confidence interval
cox_2i_: cyclooxygenase_2_ inhibitor
ERAS: enhanced recovery after surgery
NRS: Numeric Rating Scale for pain
NSAID: non-steroidal anti-inflammatory drug
OME: oral morphine equivalent
PACU: post-anesthesia care unit
PCA: patient-controlled analgesia
PONV: postoperative nausea and vomiting
QoR-15: Quality of Recovery-15 score
RR: risk ratio
THR: total hip replacement
WHODAS 2.0: World Health Organization Disability Assessment Schedule version 2.0
